# Visibility of significant prostate cancer on multiparametric magnetic resonance imaging (MRI)—do we still need contrast media?

**DOI:** 10.1007/s00330-020-07494-1

**Published:** 2020-12-02

**Authors:** Nicolai Alexander Huebner, Stephan Korn, Irene Resch, Bernhard Grubmüller, Tobias Gross, Robert Gale, Gero Kramer, Nina Poetsch, Paola Clauser, Andrea Haitel, Harun Fajkovic, Shahrokh F. Shariat, Pascal A. Baltzer

**Affiliations:** 1 Department of Urology, Medical University of Vienna, Vienna, Austria; 2 Working Group for Diagnostic imaging in Urology (ABDU), Austrian association of Urology (ÖGU), Vienna, Austria; 3 Department of Biomedical Imaging and Image-guided Therapy, Medical University of Vienna, Währinger Gürtel 18-20 1090, Vienna, Austria; 4 Department of Pathology, Medical University of Vienna, Vienna, Austria; 5 Karl Landsteiner Institute of Urology and Andrology, Vienna, Austria; 6 Division of Urology, Department of Urology, University of Jordan, Amman, Jordan; 7 Institute of Urology and Reproductive Health, Sechenov University, Moscow, Russia; 8 Department of Urology, Weill Cornell Medical Centre, New York, USA; 9 Department of Urology, University of Texas Southwestern, Dallas, USA

**Keywords:** mpMRI, Prostate cancer, Diffusion MRI, AUC, Clinical decision-making

## Abstract

**Objectives:**

To assess the visibility of clinically significant prostate cancer (PCA) lesions on the sequences multiparametric MRI of the prostate (mpMRI) and to evaluate whether the addition of dynamic contrast–enhanced imaging (DCE) improves the overall visibility.

**Methods:**

We retrospectively evaluated multiparametric MRI images of 119 lesions in 111 patients with biopsy-proven clinically significant PCA. Three readers assigned visual grading scores for visibility on each sequence, and a visual grading characteristic analysis was performed. Linear regression was used to explore which factors contributed to visibility in individual sequences.

**Results:**

The visibility of lesions was significantly better with mpMRI when compared to biparametric MRI in visual grading characteristic (VGC) analysis, with an AUC_VGC_ of 0.62 (95% CI 0.55–0.69; *p* < 0.001). This benefit was seen across all readers. Multivariable linear regression revealed that a location in the peripheral zone was associated with better visibility on T2-weighted imaging (T2w). A higher Prostate Imaging-Reporting and Data System (PI-RADS) score was associated with better visibility on both diffusion-weighted imaging (DWI) and DCE. Increased lesion size was associated with better visibility on all sequences.

**Conclusions:**

Visibility of clinically significant PCA is improved by using mpMRI. DCE and DWI images independently improve lesion visibility compared to T2w images alone. Further research into the potential of DCE to impact on clinical decision-making is suggested.

**Key Points:**

*• DCE and DWI images independently improve clinically significant prostate cancer lesion visibility compared to T2w images alone.*

*• Multiparametric MRI (DCE, DWI, T2w) achieved significantly higher visibility scores than biparametric MRI (DWI, T2w).*

*• Location in the transition zone is associated with poor visibility on T2w, while it did not affect visibility on DWI or DCE.*

**Supplementary Information:**

The online version contains supplementary material available at 10.1007/s00330-020-07494-1.

## Introduction

Multiparametric magnetic resonance imaging (mpMRI) of the prostate, consisting of at least T2-weighted imaging (T2w), diffusion-weighted imaging (DWI), and dynamic contrast–enhanced imaging (DCE) sequences, has become an important imaging modality in the diagnosis of clinically significant prostate cancer (PCA) [1]. Multiple prospective trials reported its superiority to systematic 12-core prostate biopsy alone [2, 3]. As of 2019, the European Association of Urology (EAU) guidelines recommend mpMRI to be used before prostate biopsy in patients with suspicion of PCA based on elevated prostate-specific antigen (PSA) or a suspicious digital rectal examination (DRE) [4].

These high-quality data and the adoption in guideline recommendations have led to a substantial increase in the demand for prostate mpMRI examinations with its associated increase in expenditure of time and cost. As an option to better manage the available resources, modifications in imaging protocols for prostate mpMRI have been examined. A promising such approach, referred to as biparametric MRI (bpMRI), omits the DCE sequence, thereby shortening the image capture duration and avoiding the intravenous injection of contrast agents. bpMRI has, indeed, achieved a promising negative predictive value (NPV) in a prospective trial [5]. Omitting DCE is an obvious choice, as it contributes only minimally to the clinical decision rule of the Prostate Imaging-Reporting and Data System (PI-RADS v2.1) scoring [6]: positive DCE findings upgrade peripheral zone PI-RADS 3 lesions to PI-RADS 4. This has been shown to rarely change the clinical management, as in both cases (i.e., PI-RADS 3 and 4) prostate biopsy is recommended [7]. Moreover, retrospective studies and meta-analyses have shown bpMRI to be equal to mpMRI with regard to diagnostic accuracy [8].

However, the current evidence does not account for lesion detection, as the application of PI-RADS with and without DCE data is largely a classification task. The robustness of MRI sequences is variable due to different susceptibility to, e.g., motion and air-tissue transitions. DWI as the leading sequence for the peripheral zone of the prostate is especially susceptible to artifacts, which can lead to lesions being missed. We hypothesize that maximizing the available information does increase the visibility of clinically significant PCA. This study aimed to assess the visibility of clinically significant PCA lesions on different sequences of prostate MRI and to evaluate whether the addition of a DCE sequence improves upon the overall visibility.

## Materials and methods

### Patients

This was a retrospective single-center analysis and informed consent was waived by the local ethics committee. We evaluated all consecutive patients between 2017 and 2019 referred to MRI-ultrasound-fusion biopsy for suspicious MRI, defined as PI-RADS 3 to 5 at our center. Patients were collected in a prospectively populated database. We then identified all patients with targeted biopsy-confirmed significant prostate cancer, defined as an International Society of Urologic Pathology prognostic group (ISUP) of equal to or greater than 2. The mpMRI examinations of these patients were retrospectively re-analyzed for this study. Patients were excluded if one or more sequences were not available to be evaluated or of insufficient quality due to movement or interference artifacts. One hundred nineteen lesions in 111 consecutive patients were included into the final analysis. Details are given in the flowchart (supplemental material 1) and table (supplemental material 2).

### Imaging and image-guided biopsy

All patients included in the analysis had undergone mpMRI at 8 different referring and in-house sites including T2w, DWI, and DCE sequences, all protocols being in line with technical PI-RADS recommendations (details given in supplemental material 2, second table) and graded according to the PI-RADS v2 [9] criteria, considered standard at the time. Lesion location was categorized to either the peripheral zone (PZ), transition zone (TZ), or both. They then underwent transrectal MRI-ultrasound fusion biopsy using the UroNav System (Invivo, PHILIPS©), with 3–4 additional targeted cores taken from all MRI suspicious lesions (PI-RADS 3–5) under local anesthesia. Lesions were marked previously to biopsy by a single expert radiologist with more than 10 years of experience using the DynaCAD software (Invivo, PHILIPS©).

Biopsy cores were graded according to the ISUP consensus conference 2014, by a dedicated uro-pathologist. The cores taken from a target lesion were graded as a singular sample. Our pathologist graded and reported all 3–4 cores taken from the targeted region as one singular core. This means for 4 cores taken as targeted biopsy, the report would be, e.g., Gleason 4 + 3/ISUP3 in 65% of a 46-mm sample. Clinical data was collected on PSA levels within 4 weeks prior to biopsy, DRE, prostate volume (PVol) as measured according to PI-RADS v2 on MRI images, and the calculated PSA density (PSAD).

### Image analysis

The images of all included patients were retrospectively reviewed at our center, and lesion visibility was assigned a visual grading (VG) score on a scale from 1 to 5 as follows: 1 poor/non-diagnostic, 2 poor—but still interpretable, 3 acceptable, 4 good, and 5 excellent.

Scoring was done on every sequence included in the mpMRI (T2w, DWI, DCE) separately, and the highest value achieved was used for comparison between mpMRI and bpMRI. Visibility of lesions on DWI image data was performed using both high *b*-value and ADC map together and assigning one score. E.g., poor visibility on high *b*-value images and good visibility on the ADC map could have yielded an “acceptable” visibility on DWI rating. This was not predefined but left at the respective reader’s discretion. To make sure the results were reproducible, three independent readers of varying levels of expertise as defined by the combined consensus statement on mpMRI by the European Society of Urogenital Radiology (ESUR) and the EAU Section of Urologic Imaging (ESUI) [10] (R1 radiology resident—less than basic experience in prostate MRI; R2 urologist—basic experience with prostate MRI; R3 senior faculty radiologist specialized in MRI with expert experience in prostate MRI) graded every sequence.

### Statistical analysis

Continuous data are presented as median and interquartile range, categorical as numbers and percentages. The primary endpoint of the study was the difference in visibility between mpMRI and bpMRI. We performed a visual grading-characteristics (VGC) analysis, a non-parametric rank-invariant statistical method derived from receiver operating characteristic (ROC) analysis [11], using the previously assigned score of visibility. The highest of the three scores for mpMRI and the highest of two scores for bpMRI were used for the comparison of every reviewer. The mean value out of the three reviewers was used for the primary analysis to control for inter-observer variability. A VGC curve was plotted with bpMRI on the *x*- and mpMRI on the *y*-axis. As an area under the curve (AUC) of 0.5 represents equality of the two modalities, a 95% confidence interval not crossing the 0.5 threshold was considered statistically significant. The result was labeled AUC_VGC_. For secondary analysis, we performed VGC analysis for each reviewer individually as well as comparisons between sequences. VGC analysis and curves were generated using non-parametric ROC analysis. Inter-reader agreement was assessed using Fleiss’ kappa.

In further exploratory analysis, we investigated clinical factors that might affect visibility on individual sequences of the MRI. As we used combined data for all three reviewers, and residuals were following normal distribution after testing of skewness and kurtosis, linear regression was used as opposed to ordered regression. We performed univariable linear regression analyses to assess visibility on each individual sequence and to evaluate for an association with clinical data. Subsequently, a multivariable linear regression model was built.

Due to the exploratory nature of our study, statistical significance was considered for *p* < 0.05, but not in a confirmatory matter. Thus, no adjustments for multiplicity were performed. All tests were two-sided, and all analysis were conducted using STATA (StataCorp).

## Results

### Baseline characteristics and VGC analysis

The baseline characteristics of the lesions included in our final analysis are shown in Table 1 (further details in supplemental material 1). The time between MRI and biopsy ranged between 0 and 6 months while the time between PSA measurement and biopsy ranged between 0 and 4 weeks. None of the patients underwent prostate biopsies within 6 weeks prior to the MRI. Median PSA was 7.9 ng/ml with a median PVol of 32.6 cc. The median lesion size as determined by measuring the longest diameter on axial images was 14 mm. Only 3.4% were graded PI-RADS 3 on the initial radiological report with the rest being PI-RADS 4 (43.7%) and 5 (52.9%). Lesions were located in the PZ in 77.3% of cases, in the TZ in 18.5%, and in both zones in 4.2%. According to the ISUP classification, there were 42.0%, 26.1%, 16.8%, and 15.1% of lesions graded as grade group 2, 3, 4, and 5, respectively. As our definition of clinically significant PCA started at ISUP 2, there were no ISUP 1 lesions.

**Table 1:** Baseline characteristics of 119 prostate lesions in 111 consecutive patients with clinically significant prostate cancer on MRI-ultrasound fusion biopsy

	Median	IQR (P25–P75)
Age (years)	71	64–77
PSA (ng/ml)	7.9	5.47–12
Prostate volume (cc)	32.6	25.1–46.4
PSA density	0.24	0.16–0.38
Lesion size (mm)	14	11–18
	*N*	Percent
ISUP		
ISUP 2	50	42.0
ISUP 3	31	26.1
ISUP 4	20	16.8
ISUP 5	18	15.1
PI-RADS		
PI-RADS 3	4	3.4
PI-RADS 4	52	43.7
PI-RADS 5	63	52.9
Zone		
Peripheral zone	92	77.3
Transition zone	22	18.5
Both	5	4.2

*PSA* prostate-specific antigen, *ISUP* International Society of Urologic Pathology Grade Group, *PI-RADS* prostate imaging reporting and data system

For all three observers combined, the median VG score was 3.60 (± 0.10) in T2w, 4.20 (± 0.09) in DWI, and 4.25 (± 0.8) in DCE. A detailed comparison of sequences and reviewers is shown in Table 2.

**Table 2:** Visibility grading by 3 reviewers of 119 lesions on prostate mpMRI with biopsy-confirmed, clinically significant prostate cancer

Visibility, *n* (%)	1	2	3	4	5
Reader 1					
T2w	9 (7.5)	10 (8.4)	28 (23.5)	27 (22.7)	45 (37.9)
DWI	5 (4.2)	4 (3.4)	9 (7.5)	25 (21.0)	76 (63.9)
DCE	4 (3.4)	6 (5.0)	13 (10.9)	21 (17.7)	75 (63.0)
Reader 2					
T2w	13 (10.9)	13 (10.9)	39 (32.8)	27 (22.7)	27 (22.7)
DWI	7 (5.9)	10 (8.4)	24 (20.2)	31 (26.0)	47 (39.5)
DCE	1 (0.8)	7 (5.9)	31 (26.0)	29 (24.4)	51 (42.9)
Reader 3					
T2w	5 (4.2)	14 (11.7)	29 (24.4)	34 (28.6)	37 (31.1)
DWI	3 (2.5)	4 (3.4)	10 (8.4)	32 (26.9)	70 (58.8)
DCE	2 (1.7)	5 (4.2)	13 (10.9)	22 (18.5)	77 (64.7)

*T2w* T2-weighted imaging, *DWI* diffusion-weighted imaging, *DCE* dynamic contrast–enhanced imaging

**Fig. 1:**
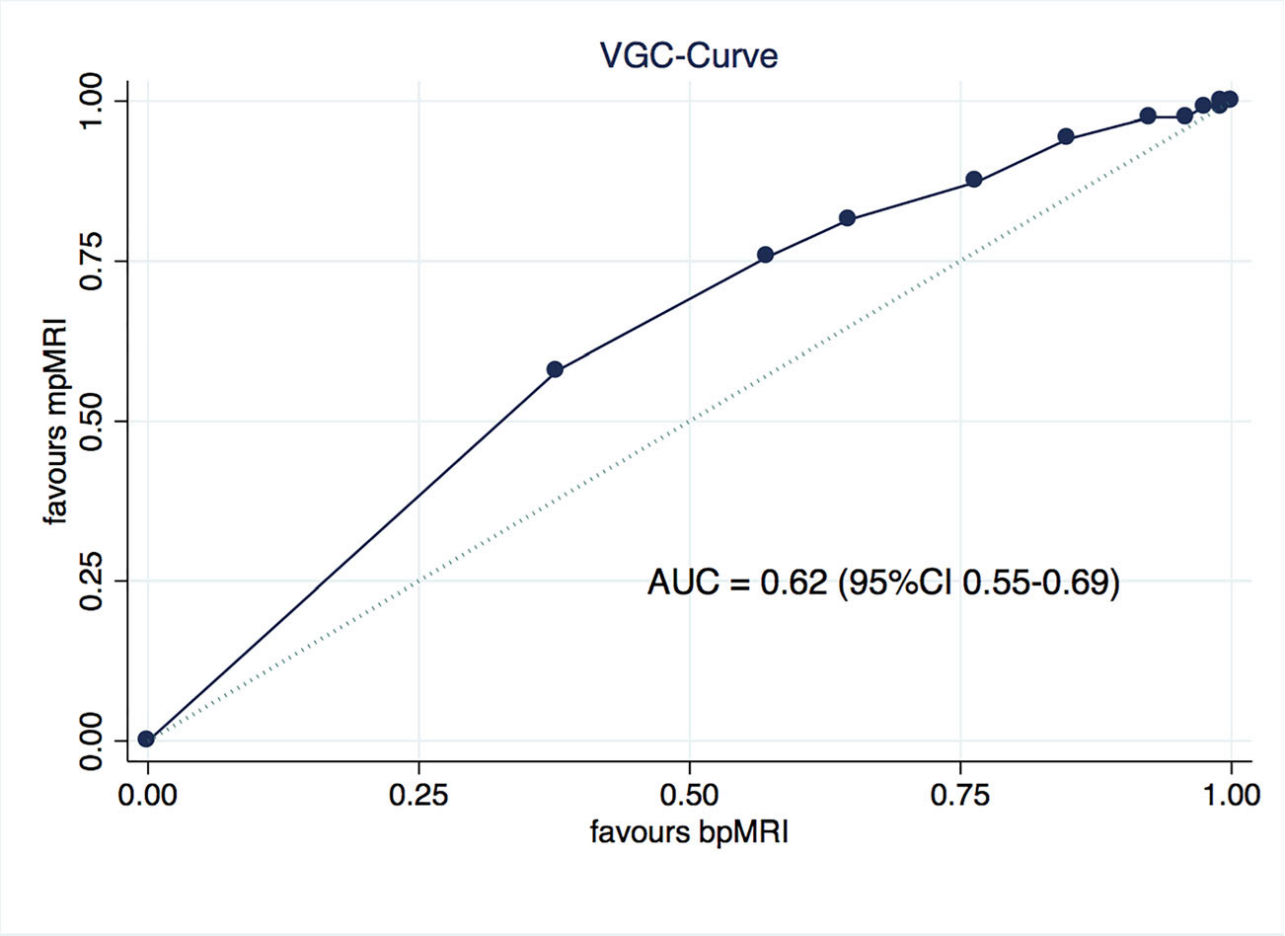
VGC curve of mpMRI vs bpMRI for 3 observers combined, obtained by non-parametric ROC analysis

**Fig. 2:**
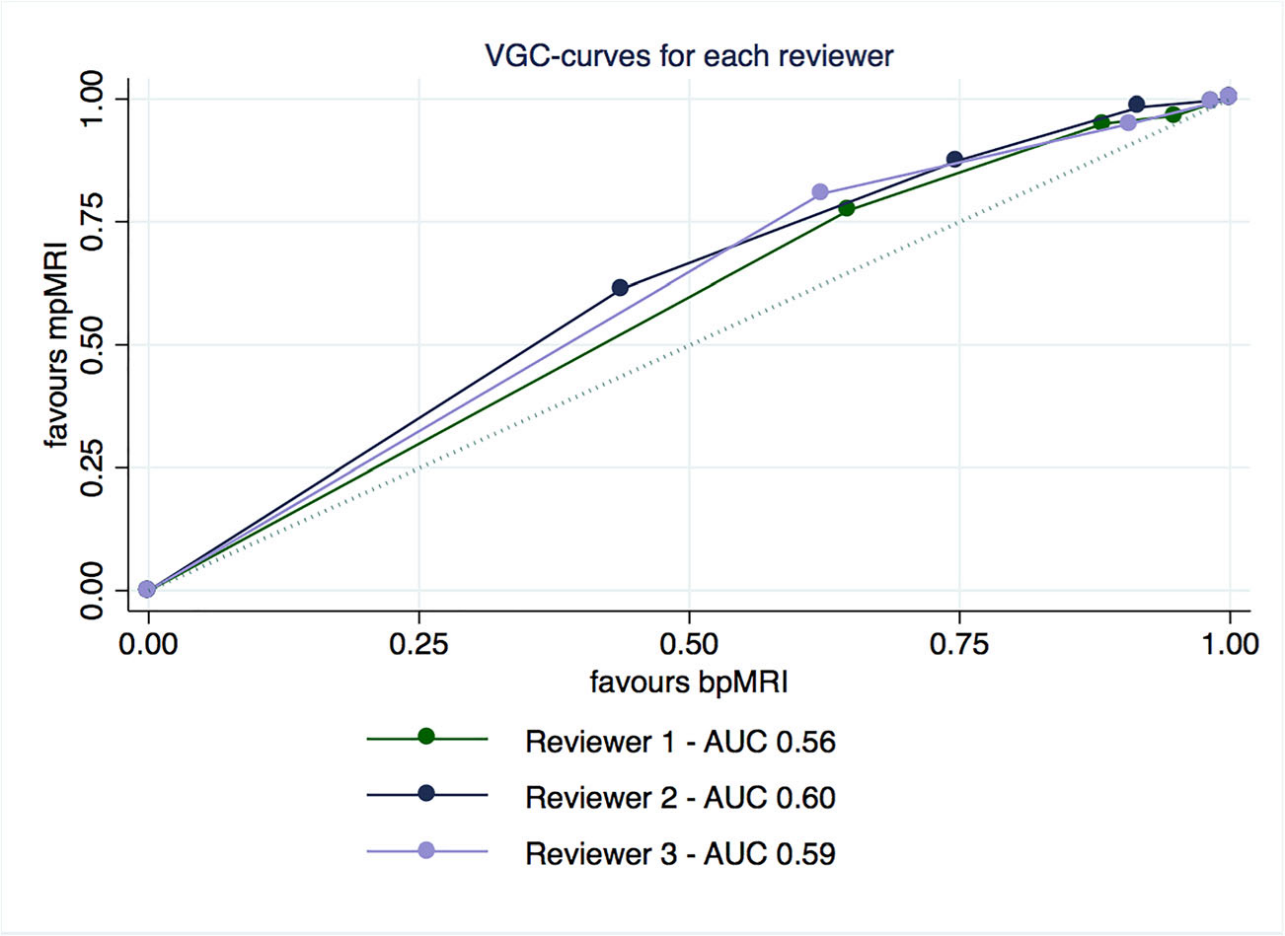
VGC curves for all individual observers, obtained by non-parametric ROC analysis

The visibility of lesions was significantly better on mpMRI when compared to bpMRI in our VGC analysis with an AUC_VGC_ of 0.62 (95% CI 0.55–0.69; *p* < 0.001) as seen in Fig. 1. This benefit was seen across all reviewers as for each reviewer individually the AUC_VGC_s were 0.56 (95% CI 0.51–0.62; *p* = 0.024) for R1, 0.60 (95% CI 0.54–0.67; *p* = 0.002) for R2, and 0.59 (95% CI 0.54–0.65; *p* = 0.002) for R3. The individual curves are depicted in Fig. 2. Inter-reader agreement between all three reviewers was acceptable given the five-point scale with a kappa of 0.36, 0.38, and 0.41 for T2w, DWI, and DCE, respectively.

In VGC analysis of individual sequences, DWI as well as DCE showed better visibility than T2w with an AUC_VGC_ of 0.67 (95% CI 0.60–0.73; *p* < 0.001) for DWI and 0.68 (95% CI 0.62–0.75; *p* < 0.001) for DCE. There was no significant difference between DWI and DCE with an AUC_VGC_ of 0.52 (95% CI 0.45–0.60; *p* = 0.50).

### Exploratory regression analysis of factors influencing visibility

In univariable linear regression analyses, ISUP 5 histologic grade, PI-RADS 5, and location in the PZ as well as larger lesion size were associated with improved visibility on T2w sequence. Only location in PZ (*p* < 0.001) and lesion size (*p* = 0.004) remained independently associated with improved visibility on T2w in a multivariable linear regression model adjusted for the effects of ISUP, PI-RADS, zone, and lesion size (*R*^2^ = 27%; *p* < 0.001). Location in the peripheral zone had the biggest impact with a regression coefficient of 0.94 (95% CI 0.50–1.37; *p* < 0.001) (Table 3).

**Table 3:** Uni- and multivariable linear regression analyses assessing the impact of clinical factors on the visibility of significant prostate cancer lesions by three reviewers on T2-weighted sequence of prostate MRI

T2w sequence				
	Univariable		Multivariable	
	Coefficient (95% CI)	*p*	Coefficient (95% CI)	*p*
Age (years)	− 0.00 (− 0.03 to 0.02)	0.90	–	
PSA (ng/ml)	0.01 (− 0.00 to 0.01)	0.053	–	
Prostate volume (cc)	− 0.00 (− 0.02 to 0.01)	0.63	–	
PSA density	0.26 (− 0.01 to 0.53)	0.06	–	
ISUP 2 (baseline)				
3	0.08 (− 0.41 to 0.57)	0.75	− 0.29 (− 0.75 to 0.18)	0.23
4	0.47 (− 0.10 to 1.04)	0.10	0.48 (− 0.03 to 1.00)	0.07
5	0.68 (0.09 to 1.27)	0.02	0.41 (− 0.15 to 0.98)	0.15
PI-RADS 3 (baseline)				
4	0.59 (− 0.51 to 1.69)	0.29	0.33 (− 0.69 to 1.35)	0.51
5	1.11 (0.02 to 2.21)	0.04	0.67 (− 0.39 to 1.72)	0.21
Zone peripheral (baseline)				
Transitional	− 0.66 (− 1.12 to − 0.19)	0.006	− 0.94 (− 1.37 to − 0.50)	< 0.001
Lesion size (mm)	0.05 (0.02 to 0.08)	0.001	0.05 (0.02 to 0.08)	0.004
Multivariable model adjusting for ISUP, PI-RADS, zone, and lesion size: *R*^2^ = 27%; *p* < 0.001				

*PSA* prostate-specific antigen, *ISUP* International Society of Urologic Pathology Grade, *PI-RADS* prostate imaging reporting and data system

For DWI sequence, ISUP 5, PI-RADS (4 and 5), and lesion size were associated with improved visibility on univariable linear regression analyses. Only PI-RADS and lesion size retained a significant association on multivariable linear regression analyses that adjusted for the effects of ISUP, PI-RADS, and lesion size (*R*^2^ = 29%; *p* < 0.001). For DWI, the most influential factor was PI-RADS with both PI-RADS 4 and 5 showing a coefficient of 1.62 (95% CI 0.78–2.47; *p* < 0.001) and 1.76 (95% CI 0.89–2.63; *p* < 0.001) (Table 4). DCE showed similar results to DWI, in addition to a significant association with age on univariable linear regression analyses (coefficient 0.02; 95% CI 0.00–0.04; *p* = 0.049). Yet, on multivariable analyses that adjusted for the effects of age, ISUP, PI-RADS, and lesion size, only PI-RADS and lesion size (*R*^2^ = 23%; *p* < 0.001) remained significant (Table 5).

**Table 4:** Uni- and multivariable linear regression analyses assessing the impact of clinical factors on the visibility of significant prostate cancer lesions by three reviewers on diffusion-weighted sequence of prostate MRI

DWI sequence				
	Univariable		Multivariable	
	Coefficient (95% CI)	*p*	Coefficient (95% CI)	*p*
Age (years)	0.01 (− 0.00 to 0.03)	0.14	–	
PSA (ng/ml)	0.00 (− 0.00 to 0.01)	0.15	–	
Prostate Volume (cc)	0.00 (− 0.01 to 0.02)	0.35	–	
PSA density	0.15 (− 0.08 to 0.38)	0.19	–	
ISUP 2 (baseline)				
3	0.31(− 0.11 to 0.72)	0.14	0.02 (− 0.36 to 0.40)	0.92
4	0.29 (− 0.19 to 0.77)	0.23	0.12 (− 0.31 to 0.55)	0.58
5	0.58 (0.08 to 1.08)	0.02	0.14 (− 0.32 to 0.62)	0.53
PI-RADS 3 (baseline)				
4	1.79 (0.92 to 2.65)	< 0.001	1.62 (0.78 to 2.47)	< 0.001
5	2.19 (1.33 to 3.05)	< 0.001	1.76 (0.89 to 2.63)	< 0.001
Zone peripheral (baseline)				
Transitional	0.30 (− 0.10 to 0.70)	0.15	–	
Lesion size (mm)	0.06 (0.03 to 0.08)	< 0.001	0.04 (0.02 to 0.07)	0.001
Multivariable model adjusting for ISUP, PI-RADS, and lesion size: *R*^2^ = 29%; *p* < 0.001				

*PSA* prostate-specific antigen, *ISUP* International Society of Urologic Pathology Grade, *PI-RADS* prostate imaging reporting and data system

**Table 5:** Uni- and multivariable linear regression analyses assessing the impact of clinical factors on the visibility of significant prostate cancer lesions by three reviewers on contrast–enhanced sequence of prostate MRI

DCE sequence				
	Univariable		Multivariable	
	Coefficient (95% CI)	*p*	Coefficient (95% CI)	*p*
Age (years)	0.02 (0.00 to 0.04)	0.049	0.01 (− 0.01 to 0.03)	0.22
PSA (ng/ml)	0.00 (− 0.00 to 0.01)	0.14	–	
Prostate volume (cc)	− 0.01 (− 0.01 to 0.01)	0.52	–	
PSA density	0.18 (− 0.05 to 0.40)	0.12	–	
ISUP 2 (baseline)				
3	0.46 (0.06 to 0.86)	0.02	0.20 (− 0.19 to 0.59)	0.32
4	0.22 (− 0.25 to 0.68)	0.36	0.08 (− 0.35 to 0.52)	0.70
5	0.61 (0.13 to 1.09)	0.01	0.19 (− 0.29 to 0.68)	0.44
PI-RADS 3 (baseline)				
4	1.22 (0.35 to 2.09)	0.006	1.00 (0.14 to 1.86)	0.02
5	1.66 (0.80 to 2.53)	< 0.001	1.12 (0.23 to 2.02)	0.01
Zone peripheral (baseline)				
Transitional	− 0.04 (− 0.43 to 0.36)	0.86	–	
Lesion size (mm)	0.05 (0.03 to 0.08)	< 0.001	0.04 (0.01 to 0.07)	0.002
Multivariable model adjusting for age, ISUP, PI-RADS, and lesion size: *R*^2^ = 23%; *p* < 0.001				

*PSA* prostate-specific antigen, *ISUP* International Society of Urologic Pathology Grade, *PI-RADS* prostate imaging reporting and data system

## Discussion

**Fig. 3:**
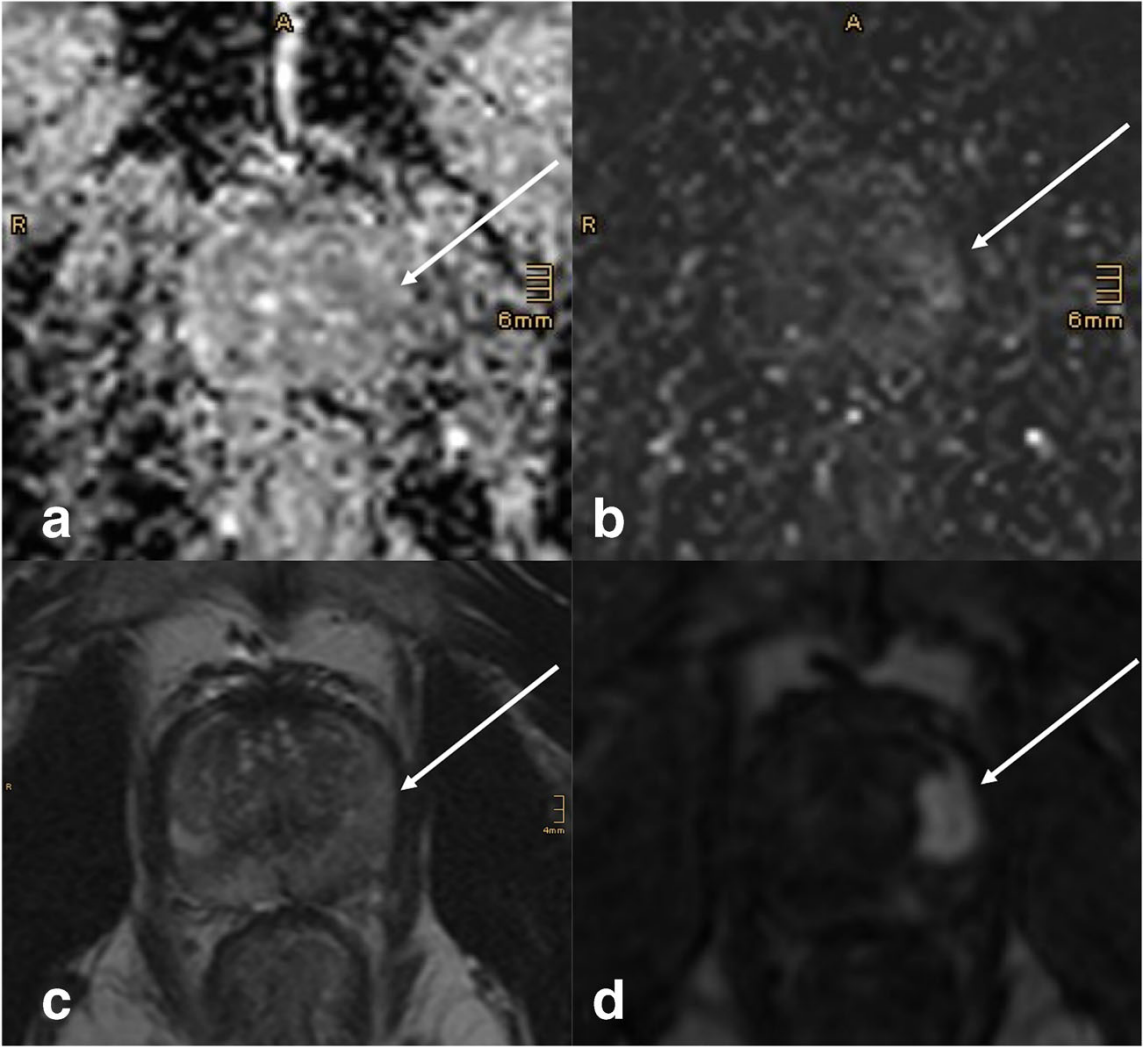
51-year-old man with a biopsy-proven ISUP 5 invasive prostate cancer visible on multiparametric MRI (white arrows). The lesion measures > 15 mm and shows only mild changes on DWI (**a** ADC map, **b** high *b*-value) and T2w (**c**). With poor visibility, the lesion might have been missed on biparametric MRI. Lesion visibility, however, is excellent on early DCE (**d**), and allows for lesion detection, and subsequent grading according to PI-RADS criteria

**Fig. 4:**
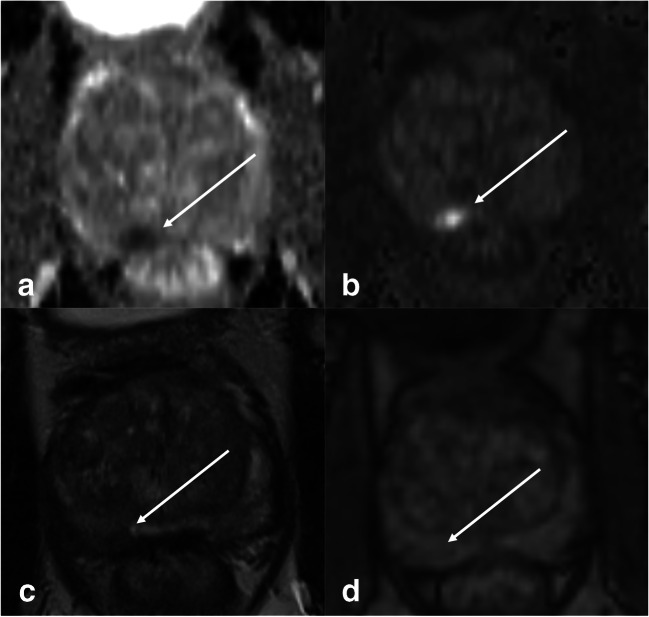
72-year-old man with a biopsy-proven ISUP 2 invasive prostate cancer visible on multiparametric MRI (white arrows). The lesion measures < 15 mm and shows typical PI-RADS 4 features on DWI (**a** ADC map, **b** high *b*-value); the visibility is excellent. On T2w (**c**), lesion visibility is acceptable while on early DCE (**d**), the lesion presents with poor visibility and would not have been detected

**Fig. 5:**
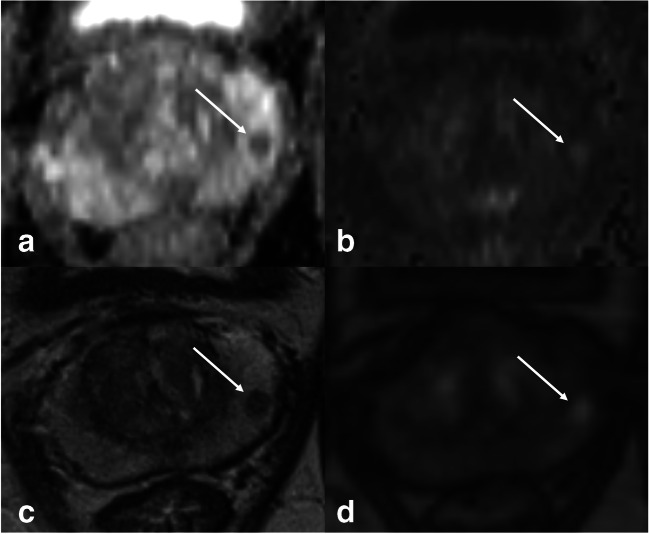
71-year-old man with a biopsy-proven ISUP 2 invasive prostate cancer visible on multiparametric MRI (white arrows). The lesion measures < 15 mm and was classified as PI-RADS 4 with good visibility on all sequences

In this study, we assessed lesion visibility of clinically significant PCa. We found significantly improved visibility scores for mpMRI over bpMRI. These scores were very similar for DWI and DCE, both being superior to T2w, yet they were not equally distributed, thus leading to an incremental visibility if used in combination. Examples of this are shown in Figs. 3, 4, and 5.

PI-RADS is a clinical decision rule based on predefined diagnostic criteria. It is used to classify visible lesions on MRI into categories based on their likelihood of harboring PCA and has been proven to be a good predictor of PCA being present [12]. Many smaller studies [13–16] and a recent systematic review including 77 studies [17] have reported varying levels of inter-reader agreement, ranging from low to almost perfect, using different versions of the PI-RADS criteria. This suggests a considerable variation in reporting of prostate MRI. Even in highly specialized centers, there is substantial inter-reader variability, as shown in the prospective PROMIS trial, where radiologist only agreed in 80% of cases, with a kappa of 0.5, whether an MRI should be considered positive (PI-RADS 3, 4, 5) or negative (PI-RADS 1, 2) [2]. These differences are unlikely to decrease when increasing mpMRI availability while reducing the parameters being provided: lesions need to be detected and therefore seen, before they can be classified. According to the literature, the PI-RADS classification does entail a non-negligible amount of false-negative result [18, 19]. However, these false negatives are often due to the potentially visible lesion being missed on the initial review as shown by Borofsky et al, where 42% of missed lesions were characterized as PI-RADS 3, 4, or 5 upon a second review [20]. This fuels the hypothesis that improved visibility improves the routine clinical accuracy of prostate MRI. Our assessment of visibility suggests a potential advantage regarding lesion detection when combining DWI and DCE data. This potential advantage may, however, yield a higher number of false-positive findings, thereby undermining the potential advantage of DCE images. Currently, the lack of data does not allow definite conclusions and a dedicated diagnostic study would be necessary to clarify the role of DCE for lesion detection.

The main driver behind bpMRI protocols is to allow MRI of the prostate to be a more widely available examination. And while this is beneficial for all patients, the wider availability will require more readers, who will likely not be specialists in the examination. While bpMRI has shown good results in two prospective trials [5, 21] with an NPV of 90% and 97% which is comparable to mpMRI in similar trials [2], there are no prospective trials directly comparing mpMRI and bpMRI. The biggest retrospective analysis is a systematic review by Woo et al [8] including 20 studies with an overall population of 2142. They showed very similar results for sensitivity and specificity for bpMRI (0.74, 0.90) and mpMRI (0.76, 0.89). This could be referred to the application of PI-RADS v2, where the only role of DCE is to potentially upgrade peripheral zone PI-RADS 3 lesions. Therefore, it is a priori very unlikely to identify significant differences between mpMRI and bpMRI using PI-RADS results.

A possible advantage using DCE images may be gained in the assessment of the primary tumor. As the landscape of local therapy for prostate cancer is evolving, and concepts of individualized therapy, such as focal therapy (FT) [22, 23], broader application of active surveillance (AS) [24, 25], and image-guided radiation, are becoming more prevalent, the need for accurate imaging is also increasing. In these settings, better visibility and improved estimation of tumor location and size are key factors to success. When planning FT, the largest tumor diameter is chosen as the ablation zone [26], which can be different on the various sequences. Also, the imaging follow-up after FT is entirely based on functional imaging and DCE currently being used the most [26, 27] as the other sequences are unreliable after tumor ablation. For AS, again, accurate estimation of tumor volume and multifocality is important to assess progression [28]. It is of utmost importance that the value of MRI in these situations is carefully assessed in the future, and to recognize that the information given by PI-RADS alone, while a great tool, is sometimes insufficient.

In our exploratory regression analysis, we found visibility on T2w sequence to be negatively impacted if the lesion was located in the TZ. Generally, the sensitivity of prostate MRI is worse in the TZ, as recently shown in a large study by Wibulpolprasert et al who found a sensitivity of 58.9% vs 51.2% (*p* < 0.001) in their adjusted per-sector analysis [29]. However, visibility in DWI or DCE did not depend on lesion location in our study. While our sample size of TZ lesions was small, the results not only are statistically significant but also showed a substantial numerical visibility difference between T2w and both DCE and DWI. So, while T2w is considered the dominant sequence for classification of TZ lesions in PI-RADS, our study showed visibility to be better in DWI and DCE. Also, in contrast to T2w, visibility on DWI and DCE was independently associated with PI-RADS scoring, suggesting DWI and DCE to be the pivotal sequences for the initial reviewers of the images. Unsurprisingly, lesion size was significantly associated with visibility in all sequences, as it is also an important factor in the PI-RADS classification and an important predictive marker for PCA being present. In contrast, there was no significant association with visibility in any of the sequences and higher tumor grade.

Our study is limited by the retrospective study design, as well as the inherent statistical problems associated with visually analyzing ordinal data such as Likert scales. We carefully chose our statistical methods to minimize these and the results were robust. Of note, one of the advantages of the VGC analysis is considered its validity [11] and previous clinical studies showed similar reproducibility to our study in different settings [30, 31]. All patients had cancer and the readers were aware of this fact though not of the individual diagnosis (i.e., ISUP grade). Therefore, we cannot make any statements on sensitivity or NPV in this study. The purpose of the study was to grade the visibility of clinically significant PCA, an aim which is not impaired by this approach as this was not a diagnostic study (i.e., no detection or classification task). Additionally, our sample size is limited, and some subgroups might not be accurately represented. Finally, MR images came from a number of different MR systems. Though image contrast and quality may vary between sites, all protocols were in line with PI-RADS recommendations and the consecutive inclusion of patients largely precluded a systematic bias. It should still be noted that magnetic resonance imaging is not a push button technique and image appearance and contrast of single sequences such as DWI depend on acquisition parameters.

## Conclusion

Visibility of clinically significant PCA is improved by using mpMRI. DCE and DWI images independently improve lesion visibility compared to T2w images alone. While this is not the same as improved sensitivity or diagnostic accuracy, it does give additional information to the treating physician. For prostate cancer detection planning of local therapy as well as surveillance, the added information gained by DCE could have a potentially meaningful impact on clinical decision-making. Further research into the benefits of DCE, preferably by prospective randomized trials, is needed to better select patients and indications where it can be omitted, and before recommending a general use of bpMRI.

## Supplementary Information

ESM 1 (DOCX 2039 kb)

## Abbreviations

AS: Active surveillance
AUC: Area under the curve
bpMRI: Biparametric MRI
DCE: Dynamic contrast–enhanced imaging
DRE: Digital rectal examination
DWI: Diffusion-weighted imaging
EAU: European Association of Urology
ESUI: EAU Section of Urologic Imaging
ESUR: European Society of Urologic Radiology
FT: Focal therapy
ISUP: International Society of Urologic Pathology prognostic group
mpMRI: Multiparametric MRI
MRI: Magnetic resonance imaging
NPV: Negative predictive value
PCA: Prostate cancer
PI-RADS: Prostate Imaging-Reporting and Data System
PSA: Prostate-specific antigen
PSAD: PSA density
PVol: Prostate volume
PZ: Peripheral zone
R1: Reader 1
R2: Reader 2
R3: Reader 3
ROC: Receiver operating characteristics
T2w: T2-weighted imaging
TZ: Transition zone
VG: Visual grading
VGC: Visual grading characteristics
