# Supplementary material for: Visibility of significant prostate cancer on multiparametric magnetic resonance imaging (MRI)—do we still need contrast media?
Source: Eur Radiol. 2020 Dec 2;31(6):3754–64. doi: 10.1007/s00330-020-07494-1 (PMC8128749; doi:10.1007/s00330-020-07494-1)

Supplement 1.: Flowchart for patient inclusion into database, and selection for this retrospective analysis

Supplement 2.: Tables providing baseline characteristics of the initial and final study population, further a table summarizing the key technical parameters of the sites where the MRI scans were performed.

Supplement 3.: Example Images of another male with biopsy confirmed ISUP 2 prostate cancer visible on mpMRI. Visibility was considered excellent for DWI/ADC (a, b), acceptable for T2w (c) and good for DCE (d).

Supplement 4.: Example Images of another male with biopsy confirmed ISUP 2 prostate cancer visible on mpMRI. Visibility was considered excellent for all imaging sequences.

**Supplement 1:**


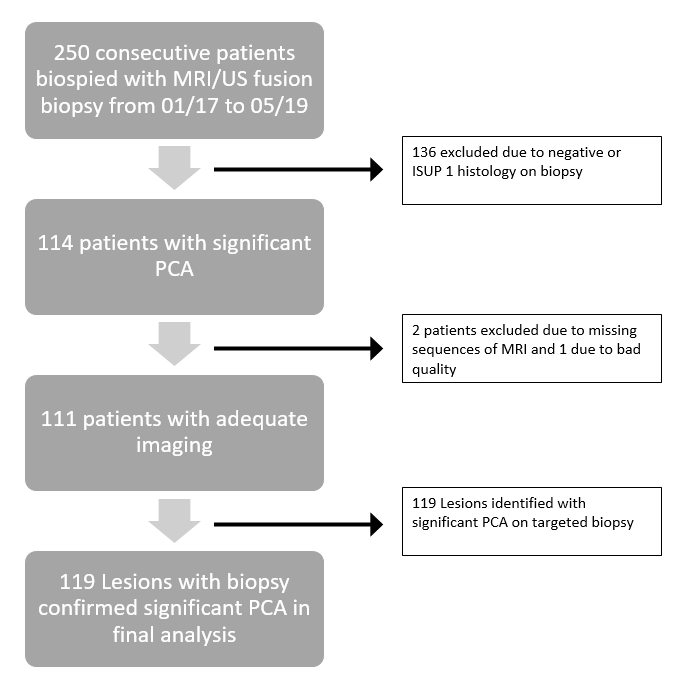


**Supplement 2:**

Baseline characteristics of 250 patients undergoing MRI Fusion Biopsy compared to 111 patients included in the final analysis

|  | **250 patients** | **Final cohort (111 patients) *** |
| --- | --- | --- |
|  | **Median (IQR)** | **Median (IQR)** |
| **Age (y)** | 67 (60-73) | 71 (64-77) |
| **PSA ng/ml** | 7.0 (4.7-10.48) | 8.0 (5.47-12) |
| **Prostate Volume (cc)** | 41.7 (29.5-60.6) | 31.7 (25.0-46.4) |
| **PSA Density** | 0.17 (0.09-0.27) | 0.25 (0.17-0.38) |
| **Lesion size (mm)** | 13.5 (11-17.5) | 14 (11-18) |
| **Time PSA to Biopsy (d)** | 4 (4-8) | 4 (4-9) |
| **Time MRI to Biopsy (d)** | 43 (29-51) | 43 (30-51) |
| **Referring center** |  |  |
| **1** |  |  |
| **2** |  |  |
| **3** |  |  |
| **4** |  |  |
| **5** |  |  |
| **XX** |  |  |
|  | | |
|  | **N (%)** | **N(%)** |
| **Previous biopsy** | 113 (45.2) | 53 (47.7) |
| **5alpha-reductase Inhibitors** | 28 (11.2) | 7 (6.3) |
| **ISUP** |  |  |
| - **0** | 89 (35.6) | 0 (0) |
| - **1** | 47 (18.8) | 0 (0) |
| - **2** | 49 (19.6) | 46 (41.5) |
| - **3** | 29 (11.6) | 29 (26.1) |
| - **4** | 18 (7.2) | 18 (16.2) |
| - **5** | 18 (7.2) | 18 (16.2) |
| **PIRADS** |  |  |
| - **3** | 46 (18.4) | 4 (3.6) |
| - **4** | 118 (47.2) | 48 (43.2) |
| - **5** | 86 (34.4) | 59(53.2) |
| **Zone** |  |  |
| - **PZ** | 201 (80.4) | 86 (77.5) |
| - **TZ** | 42 (16.8) | 20 (18.0) |
| - **both** | 7 (2.8) | 5 (4.5) |

* Note: these are numbers for the 111 patients included as opposed to the 119 individual lesions that were used for the VGC analysis, thus numbers differ to Table 1.

|  | MRI unit | T2w TR/TE (ms) | T2w FOV/matrix | DWI TR/TE (ms) | DWI b-values | DWI FOV matrix | DCE TR/TE (ms) | DCE FOV/matrix | DCE temp. res. (sec.) |
| --- | --- | --- | --- | --- | --- | --- | --- | --- | --- |
| Center 1 (n=11) | Philipps Ingenia 3T | 4000/110 | 180^2^/245*300 | 4415/74 | 0, 600, 1200 | 250^2^/84^2^ | 3.26/0.8 | 400^2^/228^2^ | 15 |
| Center 2 (n=15) | Siemens Skyra 3T | 5760/101 | 200^2^/288*320 | 5330/80 | 50, 1300, 1500 | 250^2^/112^2^ | 5.08/1.77 | 260^2^/154*192 | 8 |
| Center 3 (n=25) | Siemens Prisma fit 3T | 2880/101 | 200^2^/310*320 | 3630/48 | 0, 800, 1400* | 200^2^/118^2^ | 4.2/1.3 | 260^2^/173*192 | 6 |
| Center 4 (n=24) | Siemens Prisma fit 3T | 3800/111 | 139^2^/256*320 | 4910/60 | 0, 800, 1400* | 151^2^/ 126^2^ | 9.5/1.8 | 184^2^/138*192 | 9.5 |
| Center 5 (n=11) | Siemens Essenza 1.5T | 5900/121 | 231*224/164*320 | 5600/78 | 50, 800, 1400* | 240^2^/ 112^2^ | 4.8/1.8 | 260^2^/154*192 | 7.24 |
| Center 6 (n=11) | Siemens Vida 3T | 5700/127ms | 180^2^/307*384 | 4800/65 | 50, 1000, 1400* | 180^2^, 128^2^ | 5.1/1.8 | 240^2^/154*192 | 12.5 |
| Center 7 (n=7) | Siemens Skyra 3T | 6790/103 | 200^2^/320^2^ | 5640/59 | 50, 800, 1600* | 220^2^/ 116*118 | 5.1/1.8 | 260^2^/154*192 | 9.96 |
| Center 8 (n=7) | Siemens Skyra 3T | 6790/103 | 200^2^/ 320^2^ | 5640/59 | 50, 800, 1600* | 220^2^/116*118 | 5.1/1.8 | 260^2^/154*192 | 9.96 |

MRI units and multiparametric MRI acquisition parameters for the 111 MR examinations of the final study population

FOV given in mm, *: calculated b-value

**Supplement 3:**


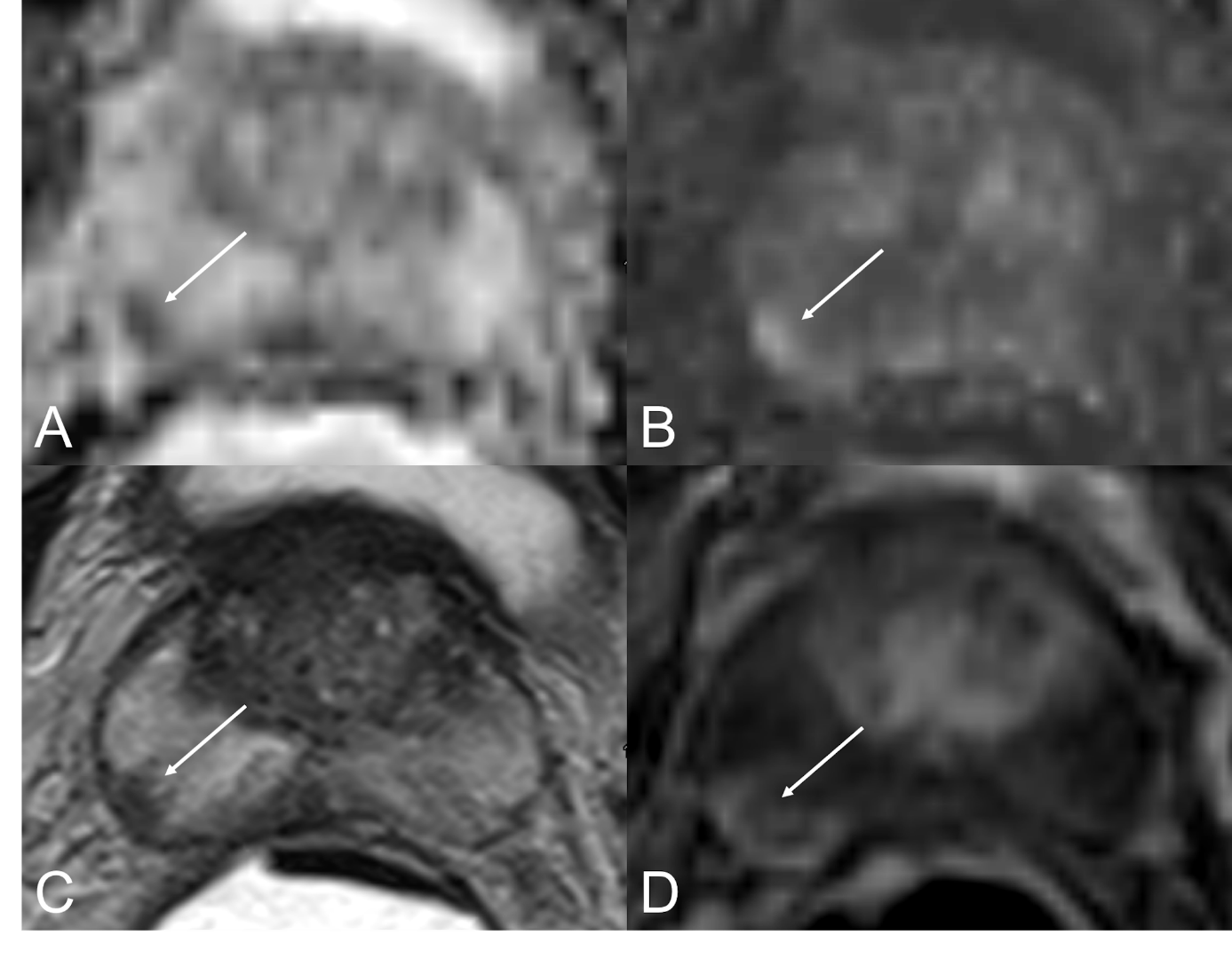


**Supplement 4:**


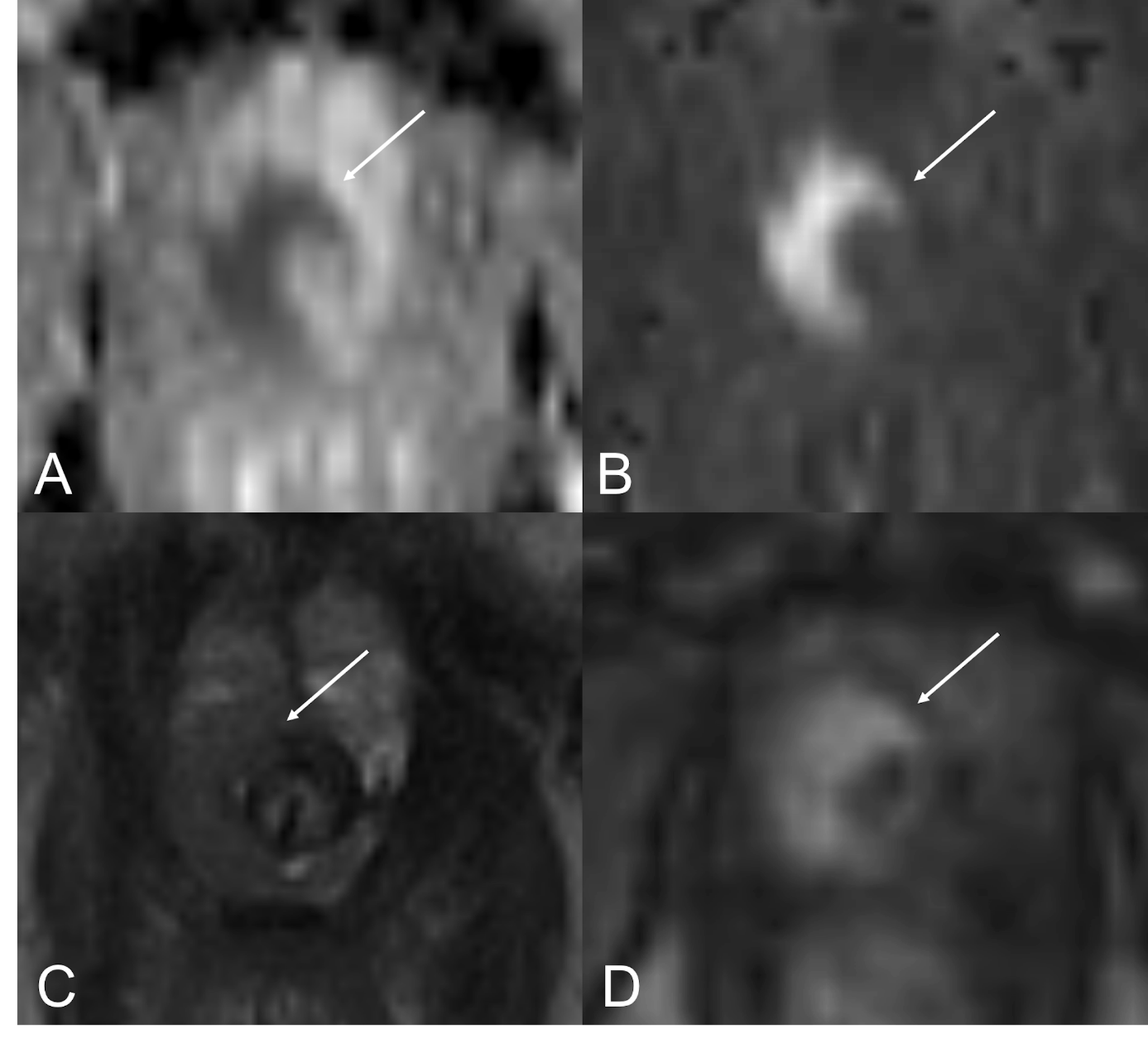

Supplement: Supplementary file 1 — (DOCX 2039 kb) [file 330_2020_7494_MOESM1_ESM.docx]
